# Scavenger receptor MARCO contributes to cellular internalization of exosomes by dynamin-dependent endocytosis and macropinocytosis

**DOI:** 10.1038/s41598-020-78464-2

**Published:** 2020-12-11

**Authors:** Sanae Kanno, Seishiro Hirano, Tsubasa Sakamoto, Akiko Furuyama, Hiroshi Takase, Hideaki Kato, Mamiko Fukuta, Yasuhiro Aoki

**Affiliations:** 1 Department of Forensic Medicine, Nagoya City University Graduate School of Medical Sciences, 1 Kawasumi, Mizuho-cho, Mizuho-ku, Nagoya, 467-8601 Japan; 2 Center for Health and Environmental Risk Research, National Institute for Environmental Studies, 16-2 Onogawa, Tsukuba, Ibaraki 305-8506 Japan; 3 Core Laboratory, Nagoya City University Graduate School of Medical Sciences, 1 Kawasumi, Mizuho-cho, Mizuho-ku, Nagoya, 467-8601 Japan

**Keywords:** Cell biology, Diseases, Nanoscience and technology

## Abstract

Macrophage receptor with collagenous structure (MARCO) is a scavenger receptor class-A protein that is expressed on the cell surface of macrophages. MARCO mediates binding and ingestion of unopsonized environmental particles, including nano-sized materials. Exosomes are cell-derived, nano-sized vesicles (40–150 nm) that can contain lipids, RNA, DNA, and various proteins. Exosomes play an essential role in cell-to-cell communication via body fluids. However, mechanisms for the recognition and internalization of exosomes by recipient cells remain poorly characterized. In this study, cellular association of serum-derived fluorescent exosomes and 20-nm fluorescent nanoparticles (positive control) was compared between MARCO-expressing (CHO-MARCO) and control (CHO-CT) CHO-K1 cells to examine whether MARCO expression by recipient cells mediates the cellular uptake of exosomes and environmental nanoparticles. Fluorescence microscopic studies and quantitative analyses revealed that the cellular associations of both exosomes and 20-nm nanoparticles were greater in CHO-MARCO cells than in CHO-CT cells. Exosomes and nanoparticles colocalized with green fluorescent protein (GFP)-MARCO in cells transfected with GFP-MARCO-encoding constructs . Furthermore, inhibitory studies showed that actin reorganization and dynamin are involved in the MARCO-mediated cellular internalization of exosomes. These results indicated that MARCO plays a role in the uptake of exosomes.

## Introduction

Exosomes are nano-sized (40–150 nm) extracellular vesicles (EVs) secreted from many types of cells into the extracellular environment. Exosomes can contain various types of functional molecules, including lipids, RNAs including messenger RNAs (mRNAs) and microRNAs, DNA, and proteins^1^. Depending on the cellular physiological condition, exosomes transfer these molecules from host cells to recipient cells or tissues via biological fluids, such as blood, urine, saliva, bronchoalveolar lavage fluid (BALF), and milk. Indeed, exosomes have emerged as pivotal mediators of cell-to-cell communication, and have been shown to contribute to the pathogenesis of many diseases, such as cancer^2^, cardiac disease^3–5^, and neurodegenerative disease^6^. These observations have suggested that exosomes have potential for use in clinical applications, including diagnosis and therapeutic treatment. Elucidation of the mechanisms whereby exosomes enter recipient cells is important for the characterization of organ-selective pathological processes and tumor metastasis, and for the development of drug delivery systems. However, the details of exosome internalization are not fully understood.

The cellular internalization of nano-sized materials is known to occur via a two-step process. Firstly, the nano-sized materials interact with the surface of the recipient cell. Previous reports have indicated that exosomes can interact with the cell membrane via the receptor/ligand pathway^7^. Various proteins have been identified as membrane-bound receptors for the binding of exosomes to recipient cells. Secondly, nano-sized materials are internalized by the recipient cells by fusion and/or various types of endocytosis, phagocytosis or macropinocytosis^7^.

Scavenger receptors (SRs) are transmembrane proteins and members of a family of pattern recognition receptors (PRRs) involved in immunosurveillance by macrophages. Class-A SRs (SR-A), one type of SR, are displayed predominantly on tissue macrophages and macrophage subtypes such as Kupffer cells^8^. The human genome encodes 6 SR-A members, including SR-A1, SR-A1.1, SR-A3 to A5, and macrophage receptor with collagenous structure (MARCO), which also is known as SR-A6^8^. The SR-A members share unique features, including a triple-helix collagenous domain that can bind a broad range of polyanionic ligands (e.g., modified low-density lipoprotein (LDL)). However, these receptors have some differences in structure, expressed cell types and tissues, and function. MARCO is expressed on alveolar macrophages and on macrophages in other areas, such as lymph node sinuses, spleen, intestine, and thymus^9^, and on Kupffer cells in the liver^10^. MARCO mediates binding and ingestion of unopsonized environmental particles such as TiO_2_, Fe_2_O_3_, silica, carbon nanotubes^11^, and pathogens such as bacteria and viruses^9,12–14^. Furthermore, in other tissues, MARCO is effective in capturing bacteria from the bloodstream^10^. Therefore, MARCO has been shown to play a pivotal role in host defense. Although the molecular structure of the MARCO protein resembles that of SR-A1, the two molecules recognize distinct ligands^15^. Notably, MARCO recognizes negatively charged surfaces via a positively charged collagenous domain^16,17^. Our previous reports demonstrated that MARCO contributes to the cellular uptake of carboxylate-modified polystyrene particles (20 nm, 200 nm, and 1 µm), which are negatively charged and relatively hydrophilic^11,18^. It has been reported that the surface of EVs also are relatively hydrophilic and have a negative charge that facilitates interactions with positively charged materials^19^. Thus, it is of interest to know whether MARCO is involved in cellular interactions with exosomes. We have constructed Chinese hamster ovary (CHO)-K1 cells introduced T-REx system-inducible MARCO expression. The system can regulate MARCO expression in response to tetracycline, thus provides an appreciate tool to examine the effect of MARCO.

In the present study, we used serum-derived fluorescent exosomes to examine whether MARCO mediates the cellular uptake of exosomes. For comparison to exosomes, we also used carboxylate-modified fluorescent 20- and 200-nm particles as a positive control. To examine the cellular association and localization of exosomes and particles, we compared these properties between tetracycline-stimulated MARCO-expressing (CHO-MARCO) and -unstimulated (CHO-CT) CHO-K1 cells using T-REx system. Furthermore, to unravel whether the specific process contributes to exosome internalization following cellular binding via MARCO, the effects of inhibitory agents were examined in CHO-MARCO cells.

## Results

### Characterization of exosomes

**Figure 1:**
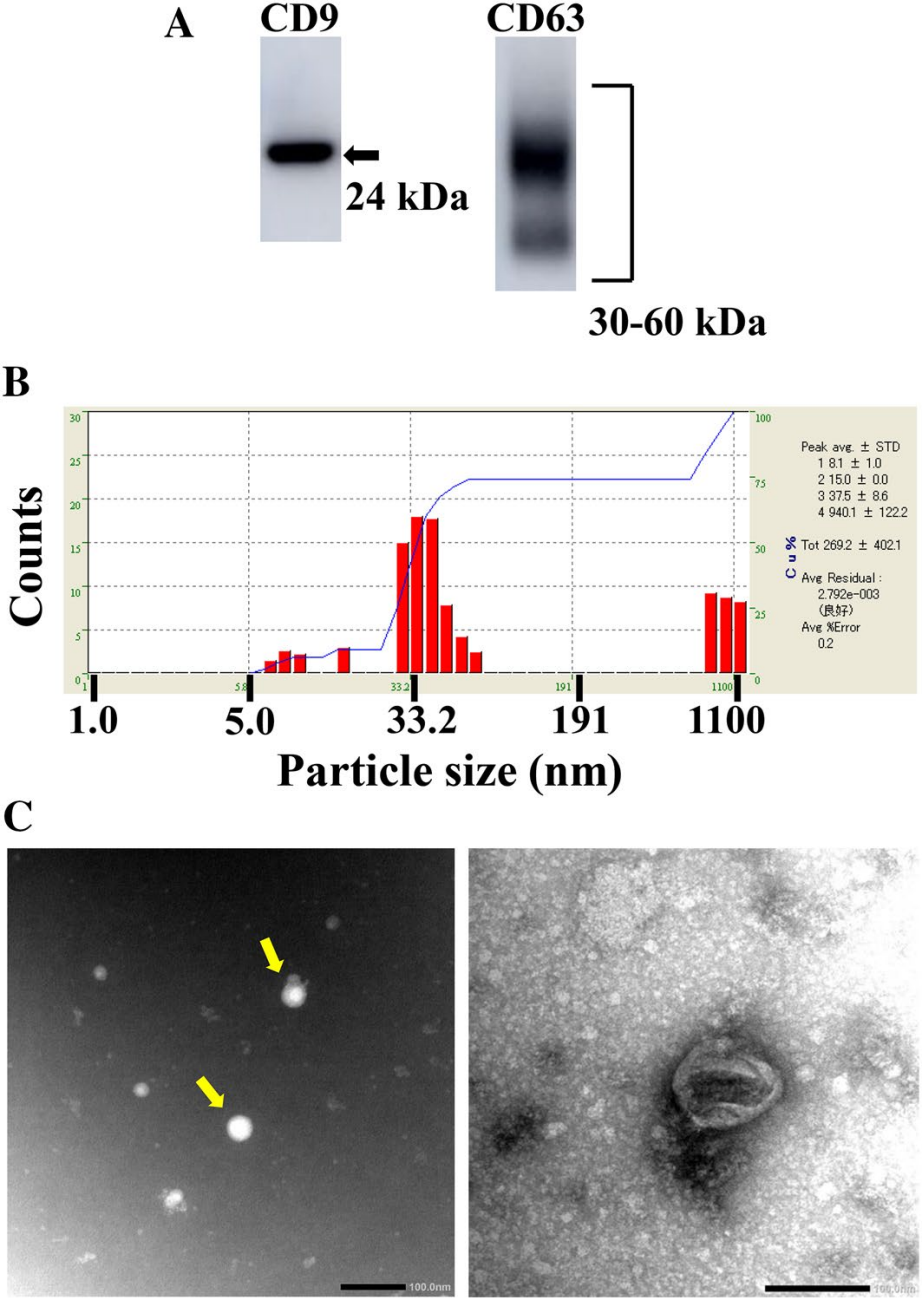
Confirmation of isolation of exosomes from human serum, using western blot analyses of CD9 and CD63 levels (**A**), size distribution (**B**), and morphology (**C**). Human serum-derived exosomes were isolated using a kit. (**A**) The lysate of the resulting exosome fraction was resolved by SDS-PAGE and electroblotted onto a PVDF membrane. The blot was probed with anti CD9- and CD63-antibodies, followed by a corresponding HRP-tagged secondary antibody. Data are representative of three experiments. Full-length blots are present in Supplemental Fig. 1. (**B**) Isolated exosomes were suspended in 100 µL PBS. The size of the exosomes was measured by dynamic light scattering. (**C**) The morphology of exosomes was examined by TEM. Scale bar = 100 nm.

Exosomal enrichment was confirmed by western blotting, size distribution, and transmission electron microscopy (TEM). As shown in Fig. 1A and supplemental Fig. 1, both CD9 and CD63 proteins, common exosomal protein markers, were detected abundantly in the lysate of serum-derived exosomes. CD63, a glycoprotein, was detected as a smeary 30- to 60-kDa band of glycosylated protein. Size distribution analyses revealed that a large number of particles with a diameter of 30–100 nm were present in the exosome fraction, as shown in Fig. 1B. We further confirmed the morphology of the isolated exosomes by TEM. TEM analyses showed that exosomes with a diameter of approximately 30 nm are present in the fraction (Fig. 1C, yellow arrows). The enlarged image (right panel) shows an exosome with a diameter about 100 nm that appears to be surrounded by lipid bilayer (Fig. 1C). These results suggested that the exosome-enriched fraction was successfully isolated from serum by this method. The exosomes obtained by the extraction methods were used for cell experiments in this study.

### Effects of MARCO on cellular association of exosomes or 20-nm nanoparticles

**Figure 2:**
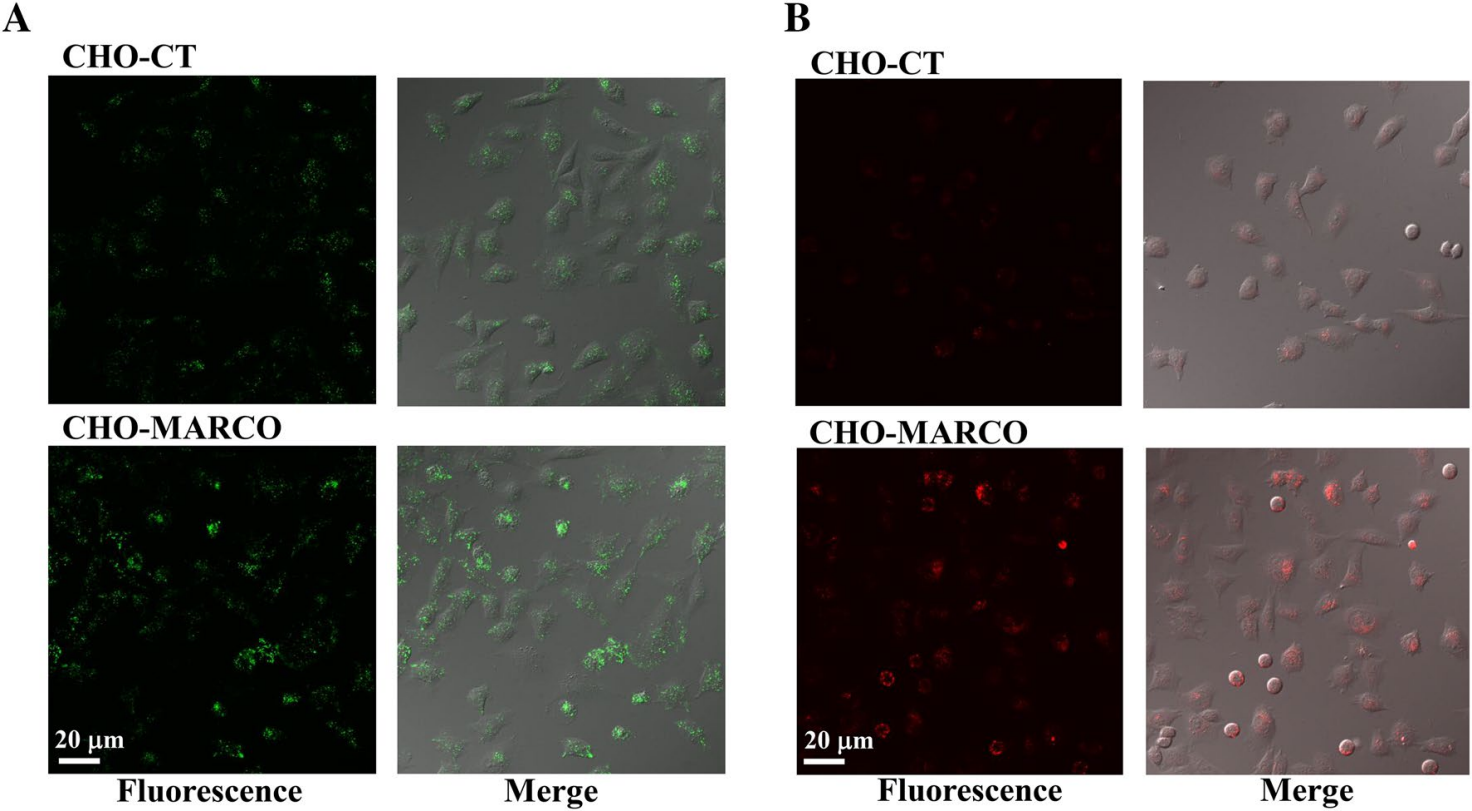
Effects of MARCO on interaction with exosomes (**A**) or 20-nm nanoparticles (**B**). T-REx-CHO-MARCO cells were cultured in a multi-well glass-bottom dish with (CHO-MARCO) or without (CHO-CT) tetracycline. After 24 h of culture, the cells were exposed to green fluorescent exosomes (**A**) or 20-nm red fluorescent nanoparticles (**B**) for 6 h. Cellular association was observed using fluorescence microscopy.

In this study, we used CHO-K1 cells stably transfected with murine MARCO (mMARCO) gene engineered into pT-REx-DEST30 Gateway Vectors (T-REx-CHO-MARCO cells). T-REx-CHO-MARCO cells were stimulated with (CHO-MARCO) or without (CHO-CT) 1 µg/mL tetracycline for 24 h to induce MARCO expression before each experiment. To examine the cellular association of exosome via MARCO, CHO-MARCO and CHO-CT cells were exposed to green fluorescent exosomes or 20-nm red fluorescent nanoparticles (positive control). The cellular associations of not only 20-nm nanoparticles (Fig. 2B), but also exosomes (Fig. 2A), were significantly increased in CHO-MARCO cells compared to CHO-CT cells. Our data on particle exposure are in accord with the results of a previously published study; particles associated with empty-vector-transfected COS-7 cells were fewer in number than those with MARCO-transfected cells^18^.

### Quantification analyses of exosomes associated with CHO-CT or CHO-MARCO cells

**Figure 3:**
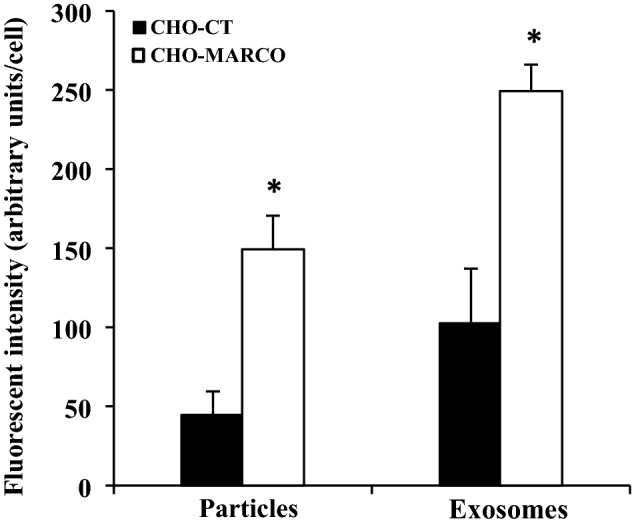
Quantification analyses of the association of exosomes or 20-nm nanoparticles with CHO-CT and CHO-MARCO cells. T-REx-CHO-MARCO cells were cultured in a 96-well plate with (CHO-MARCO) or without (CHO-CT) tetracycline. After 24 h of culture, the cells were treated with red fluorescent exosomes or 20-nm red fluorescent nanoparticles for 6 h. After incubation, the cells were washed twice with HBSS and fixed with 4% paraformaldehyde, followed by staining with DAPI and phalloidin. The associated fluorescent amounts were analyzed using an IN Cell Analyzer. Data are presented as means ± SEM (n=3). *, Significantly different from CHO-CT cells.

The fluorescence intensities of cell-associated exosomes and 20-nm nanoparticles were quantified with an IN Cell Analyzer (see “Methods”). Images used for analyses by IN Cell Analyzer are shown in Supplemental Fig. 2. As shown in Fig. 3, the fluorescence intensities of both exosomes and nanoparticles associated with CHO-MARCO cells were increased 2.5- and threefold (respectively) compared to those associated with CHO-CT cells.

### Co-localization of exosomes or 20-nm nanoparticles with MARCO

**Figure 4:**
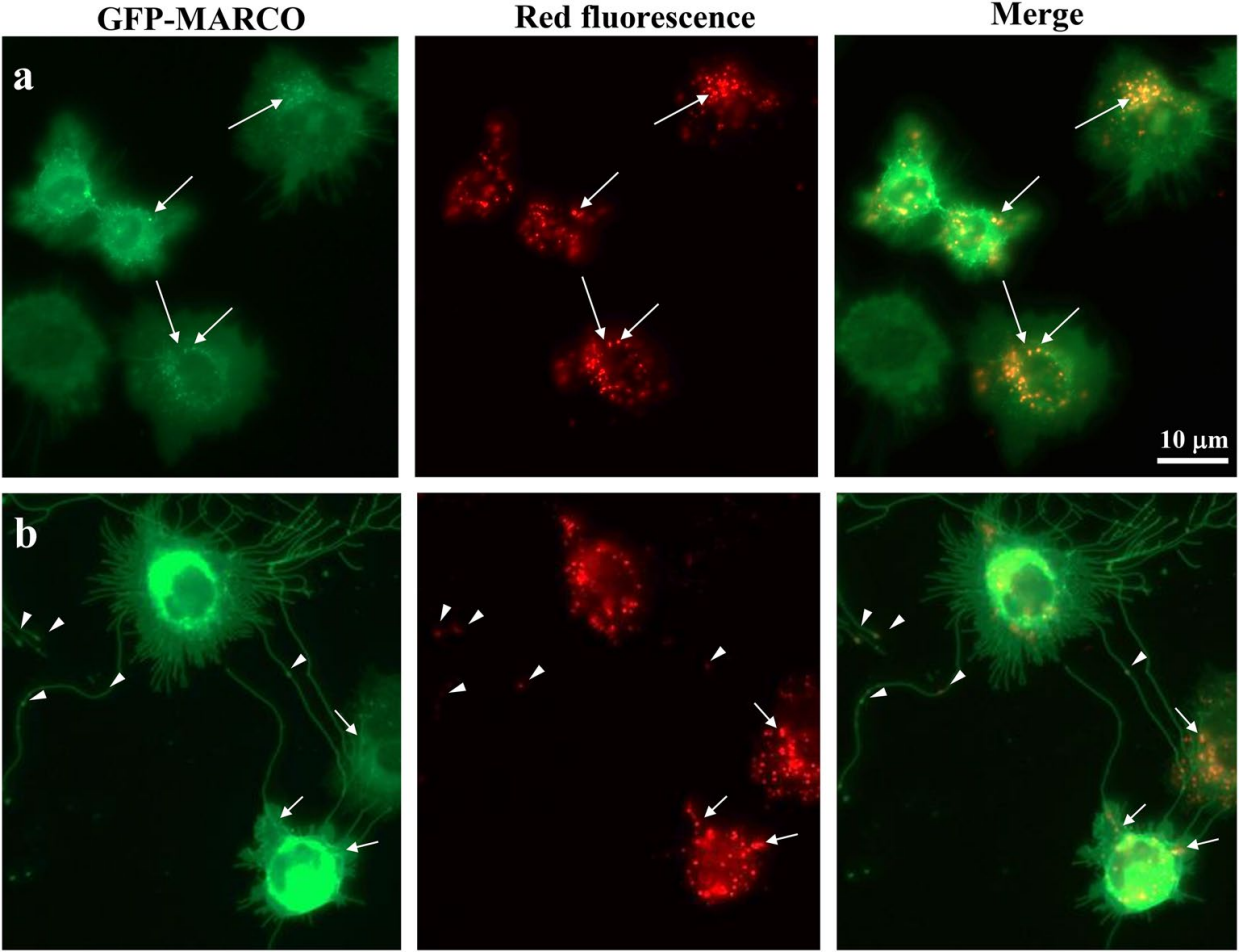
Localization of MARCO and red fluorescent exosomes (**a**) or 20-nm red fluorescent nanoparticles (**b**) in GFP-MARCO cells. GFP-MARCO cells were cultured with the red fluorescent exosomes (**a**) or 20-nm red fluorescent nanoparticles (**b**) for 6 h. The localization of MARCO and exosomes or 20-nm nanoparticles were examined using fluorescence microscopy.

CHO-K1 cells were stably transfected with green fluorescent protein (GFP)-MARCO vector (GFP-MARCO cells). To confirm the cellular localization of exosomes or 20-nm nanoparticles with MARCO, GFP-MARCO cells were used. GFP-MARCO was localized to the cell membrane and dendritic structures, as reported in previous studies^11,20^. Notably, long dendritic structures and lamellipodia-like structures were observed in GFP-MARCO cells (Fig. 4). These cell shape changes, including long dendritic and a large lamellipodia-like structures, were observed not only in ectopic MARCO-transfected CHO cells, but also in similarly transfected HeLa, NIH3T3, and 293 cells^20^. Exosomes (Fig. 4a) as well as 20-nm nanoparticles (Fig. 4b) were clearly associated with the cells and accumulated primarily in peri-nuclear regions. A portion of the exosomes and nanoparticles co-localized with the vesicles on GFP-MARCO cells, as indicated by arrows (Fig. 4a,b). In addition, 20-nm nanoparticles were associated with the dendritic structures on GFP-MARCO cells (Fig. 4b). Intriguingly, 20-nm nanoparticles also were localized at the tubercles of dendritic structures, as indicated by arrowheads. It is noteworthy that both exosomes and 20-nm nanoparticles also were associated with the lamellipodia-like structures (Fig. 4a,b, Supplemental Fig. 3, white arrows).

### Co-localization of exosomes and 20-nm nanoparticles

**Figure 5:**
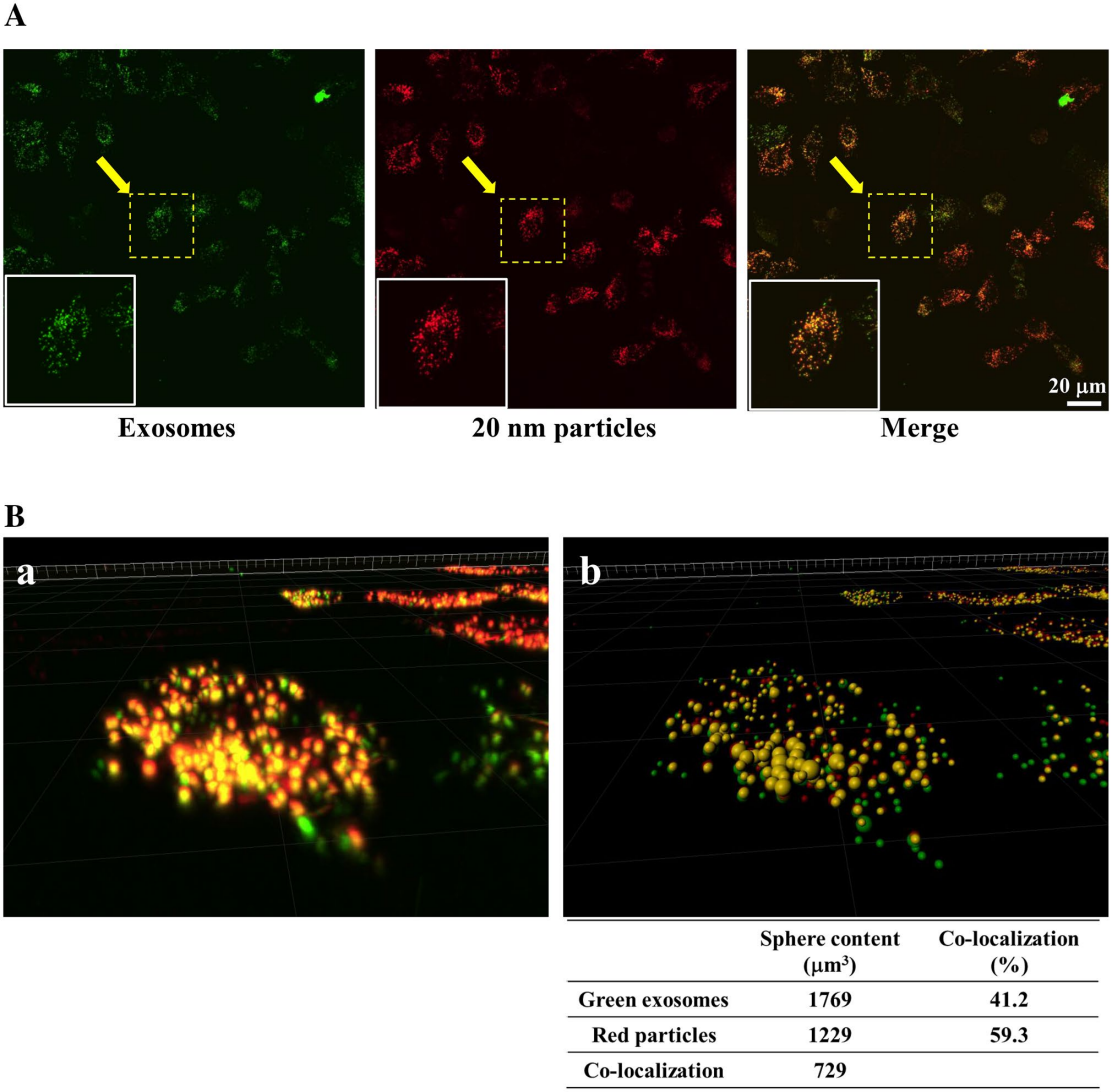
Localization of green fluorescent exosomes or 20-nm red fluorescent nanoparticles in CHO-MARCO cells. (**A**: CHO-MARCO cells were cultured with both green fluorescent exosomes and 20-nm red fluorescent nanoparticles for 6 h. The localization of exosomes and nanoparticles were observed using confocal super-resolution microscopy. (**B**) Images collected from the multiple Z-stacks were imported into the Imaris platform. The 3D image example of the region (surrounded by the dashed line) observed from the arrow in (**A**) is shown. (a) Image in 3D-renderings of raw data. (b) 3D visualization of the image using Imaris. For quantification of co-localized area, the individual sphere content of the 3D image was analyzed by Imaris using two channels, green and red. The percentage (%) was calculated as co-localized sphere content/each sphere content. The sphere volumes (µm^3^) and percentages are shown below the figure.

To examine the intracellular localization of exosomes and 20-nm nanoparticles, CHO-MARCO cells were cultured with a combination of exosomes and nanoparticles for 6 h. As shown in Fig. 5A, co-localization of exosomes (green fluorescence) and 20-nm nanoparticles (red fluorescence), indicated by yellow color (green + red overlap), indicated intracellular transfer of exosomes and 20-nm nanoparticles occurred via similar pathways. Figure 5B shows a three-dimensional (3D) image of the rectangular segment (surrounded by dashed white line) in the image of Fig. 5A; the 3D image is as viewed from the direction of the arrows. Analyses of images by the Imaris software platform revealed that approximately 41% and 60% among all the exosomes or nanoparticles taken up into the cells were co-localized in the cells respectively. In order to eliminate the possibility that exosomes adhered to 20-nm nanoparticles in the culture medium, such that the aggregates then were taken up by cells, we treated with the exosomes and nanoparticles sequentially (separated by a time lag). Fluorescence microscopic analyses revealed that whether cells were exposed first to 20-nm nanoparticles and then to exosomes (Supplemental Fig. 4a), or first to exosomes and then to 20-nm nanoparticles (Supplemental Fig. 4b), the resulting images were similar to those seen with simultaneous (combined) exposure (Fig. 5A, Supplemental Fig. 4c).

### The effects of endocytosis inhibitors on exosome internalization

**Figure 6:**
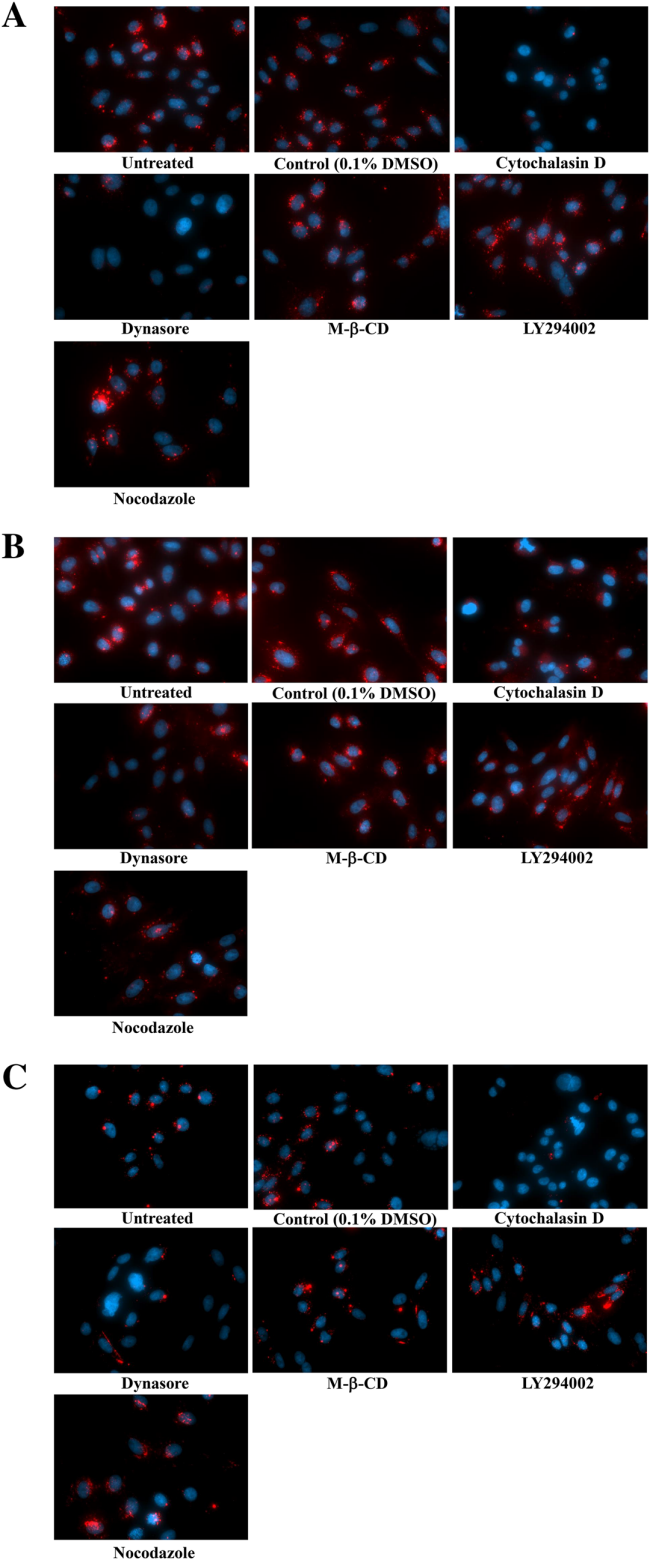
Effects of inhibitors on the internalization of exosomes (**A**), 20-nm (**B**), or 200-nm (**C**) particles. T-REx-CHO-MARCO cells were cultured with tetracycline in a 96-well black plate with clear bottom. The CHO-MARCO cells were pretreated with various inhibitors for 1 h, and then incubated with exosomes, 20-nm, or 200-nm particles in the presence of each inhibitor. After 6 h culture, the cells were washed twice with HBSS and fixed with 4% paraformaldehyde, followed by staining with DAPI. The cells were observed using fluorescence microscopy.

In order to demonstrate the contribution of specific endocytotic processes to exosome internalization following cellular binding via MARCO, we examined the effects of endocytotic inhibitors on internalization of exosomes (Fig. 6A) and compared the inhibitory effects on cells co-incubated with 20- (Fig. 6B) and 200-nm (Fig. 6C) particles. Dynasore, an inhibitor of dynamin, markedly inhibited internalization of exosomes, and of both 20- and 200-nm particles, in comparison to untreated and control cells. Treatment with cytochalasin D, a potent inhibitor of actin polymerization and reorganization, altered the morphology of CHO-MARCO cells into spheres. Cytochalasin D inhibited the uptake of both exosomes and 200-nm particles, but had only slight effects on the uptake of 20-nm nanoparticles. Notably, 20-nm nanoparticles were observed to be distributed diffusely throughout the resulting sphere-shaped cells (Fig. 6B). On the other hand, LY294002, an inhibitor of phosphatidylinositol 3-kinase (PI3K), or methyl-β-cyclodextrin (M-β-CD), an inhibitor of caveolae- and lipid raft-endocytosis, did not inhibit cellular association with exosomes or with 20- or 200-nm particles. Nocodazole, an inhibitor of microtubule polymerization, inhibited cellular association of 20-nm nanoparticles only slightly; no such inhibition was seen for exosomes or 200-nm particles. In control experiments, we confirmed that an equivalent concentration of the vehicle (solvent), 0.1% DMSO, did not affect uptake of the any of the three materials (Fig. 6A–C).

### The effects of MARCO ligand on exosome uptake

**Figure 7:**
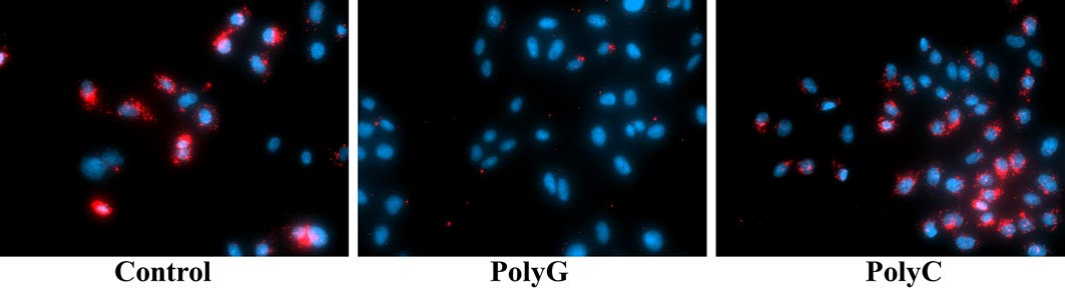
Effects of polyG or polyC on cellular association of exosomes in CHO-MARCO cells. T-REx-CHO-MARCO cells were cultured with tetracycline in a 96-well black plate with clear bottom. The CHO-MARCO cells were precultured with 200 µg/mL polyG or polyC for 30 min. The cells were exposed to red fluorescent exosomes for 4 h in the presence of each compound. See also the legend to Fig. 6 about treatments after exposure.

We investigated whether MARCO-mediated uptake of exosomes is inhibited by MARCO ligand, polyguanylic acid (polyG), in CHO-MARCO cells. PolyG significantly inhibited MARCO-mediated uptake of exosomes, whereas the negative control compound, polycytidylic acid (polyC), had no effect (Fig. 7).

## Discussion

This study was designed to examine whether the expression of MARCO in recipient cells contributed to cellular interaction with exosomes, and to investigate how cells internalize the exosomes following the interaction with MARCO. Consequently, the present studies show an important role for the MARCO receptor in internalization of exosomes, beyond MARCO’s known role in lung defense from pathogens and environmental unopsonized particles.

With regards to cellular interactions with exosomes, previous studies have identified various proteins as cell membrane receptors related to the binding of exosomes; candidates include lymphocyte function-associated antigen 1 (LFA1) on dendritic cells^21^, and T-cell immunoglobulin and mucin receptor 4 (TIM4) on phagocytes^7,22,23^. In the present study, the role of MARCO, a member of the SR-A family, in the uptake of exosomes was examined by using a tetracycline-inducible gene expression system. The association of exosomes, as well as that of 20-nm nanoparticles, were increased significantly in CHO-MARCO cells compared to CHO-CT cells (Figs. 2, 3). In GFP-MARCO cells, fluorescence microscopy analyses revealed that some exosomes were co-localized with vesicles of GFP-MARCO (Fig. 4). These results indicated that MARCO expressed in recipient cells plays a pivotal role in the uptake of exosomes. One of the most interesting findings in the present study was that MARCO functions as a membrane receptor for the binding of exosomes.

Moreover, it is worth noting that exosomes and 20-nm nanoparticles were clearly co-localized in MARCO-expressing cells (Fig. 5A). Analyses using the Imaris platform revealed that approximately 41% of exosomes and 60% of red nanoparticles among all exosomes and nanoparticles taken up by cells were co-localized within the cells, respectively (Fig. 5B). Previous studies reported that nano-sized materials ultimately localized to the lysosomal compartment^24–26^. Regarding exosomes, it has been reported that most internalized exosomes are sorted into phagolysosome and lysosomes^23,27^. Although intracellular trafficking was not investigated in the present study, we hypothesize that the exosomes and 20-nm nanoparticles co-localize within lysosomes.

Internalization of exosomes has been reported to occur via fusion and/or endocytosis^28^. Various endocytotic mechanisms for EVs, such as caveolin- or clathrin-dependent endocytosis, macropinocytosis, and lipid raft-mediated endocytosis, have been reported^29^. In the present study, screening with inhibitors indicated that dynasore markedly inhibited internalization of exosomes and of 20- and 200-nm particles (Fig. 6A–C). Dynasore is an inhibitor of dynamin, a GTPase protein that is essential for the fission of intracellular clathrin- and caveolae-coated pits^30–32^. In addition, the internalization of exosomes and of 200-nm particles, but not that of 20-nm nanoparticles, were attenuated by treatment with cytochalasin D, an inhibitor of actin polymerization and reorganization (Fig. 6A–C). In general, the endocytotic pathway largely depends on the size, shape, and charge of nano-sized materials^24,33^. Cells internalize larger particles (> 1 µm) via phagocytosis or macropinocytosis and smaller-sized particles (60–120 nm) via endocytosis^34^. The present study shows that LY294002, an inhibitor of PI3K (a protein known to be essential for phagocytosis of large particles), did not inhibit the internalization of exosomes or of 20- or 200-nm particles, suggesting that phagocytosis is not involved in the internalization of these materials. Due to their small size, exosomes, like 20- and -200 nm particles, are internalized by dynamin-dependent endocytosis. Additionally, based on morphological observations, macropinocytosis also should be associated with the internalization of exosomes in CHO-MARCO cells. Macropinocytosis, which is accompanied by membrane ruffling, is an actin-driven process that permits uptake of larger materials and extracellular non-specific fluids and proteins. It also has been reported that endothelial cell-derived exosomes^35^ or oligodendrocyte-derived exosomes^36^ are internalized by macrophages or microglia via membrane ruffling and macropinocytosis, respectively. Our study indicated that exosomes and 20-nm nanoparticles were co-localized with lamellipodia, cellular domains that exhibit membrane ruffling (Supplemental Fig. 3, white arrow). Nanoparticles of 20 nm also were observed in dendritic structures as well as the peri-nuclear region of the cell (Fig. 4b). Notably, 20-nm nanoparticles localized with the tubercles on the dendritic structures, as shown by arrowheads (Fig. 4b). In our previous report using CHO-MARCO cells, nanomaterials became tethered to the dendritic structures, and then were taken up by the cell body via micropinocytosis-like membrane ruffing while the cell body moved along the dendritic structures^11^. It has been reported that an interaction between dynamin and the actin cytoskeleton occurs during the formation of membrane ruffles and lamellipodia^37^. Membrane ruffling is inhibited by treatment with inhibitors of dynamin or of actin rearrangement^38^. Taken together, these observations suggest that macropinocytosis via membrane ruffling may be associated with the internalization by CHO-MARCO cells of exosomes as well as of nanomaterials. Previous work has demonstrated that exosomes are internalized not only via macropinocytosis but also via endocytosis. It has been reported that the cellular internalization pathway may depend on the type of recipient cell, the origin of the exosomes, and/or the environment^39^ in addition to the particle’s size, charge, and shape. Moreover, research indicates that the surfaces of nanoparticles adhere to various plasma proteins under physiological conditions, forming so-called “protein coronas”^40,41^. According to the manufacturer’s description, the carboxylate-modified nanoparticles used in the present study are expected to adhere to proteins and other biomolecules, but with extremely decreased avidity compared to those of hydrophobic particles. As with nanoparticles, the surfaces of exosomes have been reported to bind to proteins^42^. Modifications to the surfaces of particles or exosomes may affect the activity of the internalization machinery.

Exosomes have been reported to play an important role in metastasis, though the mechanisms of metastasis remain poorly understood. Organs such as liver, lung, and brain are commonly the sites not only of primary tumors, but also of metastatic growths. Exosomes have been reported to play an important role in metastasis^43^. Macrophages and Kupffer cells are key players in the uptake of exosomes and the delivery of tumor exosome messages that promote metastasis^43^. Because macrophages and Kupffer cells in lung or liver express MARCO, MARCO may be involved in tumor progression via exosome uptake. It has been reported that the majority of injected exosomes is rapidly taken up by macrophages in the liver reticuloendothelial system, irrespective of cell source^44^. Notably, the clearance of cell-derived exosomes is dramatically delayed in macrophage-depleted mice^44^. Furthermore, the uptake of tumor-derived exosomes by Kupffer cells has been shown to enhance liver metastasis^45,46^. The types of integrin present in tumor-derived exosomes are associated with organ selectivity^46^. It appears that the uptake of EVs by macrophages or Kupffer cells is involved in the enhancement of metastasis. In addition, treatment with SR-A blockers has been shown to result in decreased clearance of EVs from liver^47^. Thus, it is plausible that the EV uptake via SR-As, including MARCO, is associated with enhancement of metastasis.

A recent study attempted to enhance tumor delivery of engineered EVs using nonselective SR-A inhibitors; that work showed that inhibition of SR-A reduced the clearance of EVs from liver, resulting in increased accumulation of the engineered EVs in tumors, an effect that would enhance the therapeutic use of such EVs^47^. In future studies for the development of engineered EVs for therapeutic applications, improved efficacy might be obtained by focusing on individual SR-A members (such as SR-A1/A1.1 and MARCO) known to be expressed on phagocytes . In this context, our results are expected to provide important information for EV application in drug delivery systems.

In conclusion, we examined that cellular interaction and internalization pathways of exosomes via MARCO. The associations and fluorescent intensities of both 20-nm nanoparticles and exosomes were greater in CHO-MARCO cells than in CHO-CT cells. Fluorescent microscopic studies revealed that some exosomes, as well as 20-nm nanoparticles, were co-localized with GFP-MARCO protein in GFP-MARCO cells. Furthermore, the internalization of exosomes following binding to MARCO was mediated via dynamin-dependent endocytosis or micropinocytosis. The uptake of exosomes was inhibited by MARCO ligand, polyG, in CHO-MARCO cells. Collectively, these results indicated that MARCO in recipient cells plays a pivotal role in the uptake of exosomes.

## Methods

### Chemicals

Chemicals and reagents used in this study were obtained as follows: cytochalasin D, LY294002 and Hanks’ Balanced Salt Solution (HBSS) from Fujifilm-WAKO (Osaka, Japan); dynasore from Tokyo Kasei (Saitama, Japan); M-β-CD, nocodazole, polyG, polyC and PKH26 red- and PKH67 green-fluorescent cell linker kits from Sigma-Aldrich (St Louis, MO); anti-CD9 antibody (#sc-59140), anti-CD63 (#sc-5275) antibody, radioimmunoprecipitation (RIPA) buffer containing containing protease inhibitor, phenylmethylsulfonyl fluoride (PMSF) and sodium orthovanadate from Santa Cruz Biotechnology (Santa Cruz, CA); Polyvinylidene difluoride (PVDF) Blocking Reagent from TOYOBO (Osaka, Japan); ECL prime western detection reagent from Cytiva (Marlborough, MA); Horseradish peroxidase (HRP)-tagged anti-mouse immunoglobulin G (IgG) antibody from MBL (Nagoya, Japan); ExoQuick from System Biosciences (Palo Alto, CA); Exosome Spin Columns (MW3000), Halt phosphatase inhibitor cocktail, Lipofectamine LTX-PLUS, bicinchoninic acid (BCA) protein assay kit, DAPI, and FluoSpheres carboxylate-modified microspheres from Thermo Fisher Scientific (Waltham, MA); and phalloidin-iFluor 488 Reagent from Abcam (Cambridge, UK).

### Cell culture and transfection

Preparation of the plasmid construct was described elsewhere^11,48^. Briefly, the mMARCO-encoding gene was first engineered into pCR8/GW/TOPO and then into pT-REx-DEST30 and pcDNA 6.2/N-EmGFP using the Gateway LR Clonase Enzyme Mix (Thermo Fisher Scientific) according to the manufacturer’s instructions. CHO-K1 cells were transfected with the pT-REx-DEST30-MARCO (T-REx-CHO-MARCO cells) or GFP-MARCO (GFP-MARCO cells) vector by Lipofectamine LTX-PLUS, respectively. Stable transfectants were selected using G418 (T-REx-CHO-MARCO cells) or blasticidin-S (GFP-MARCO cells). T-REx-CHO-MARCO cells were stimulated with (CHO-MARCO) or without (CHO-CT) 1 µg/mL tetracycline for 24 h to induce MARCO expression before each experiment.

T-REx-CHO-MARCO or GFP-MARCO cells were cultured at 37 °C in a 5% CO_2_ atmosphere in F12 growth medium supplemented with 10% heat-inactivated fetal bovine serum (FBS), 100 U/mL penicillin, 100 µg/ml streptomycin, and 50 µg/mL G418 for T-REx-CHO-MARCO cells or 5 µg/mL blasticidin-S for GFP-MARCO cells. In all cell culture experiments, medium containing 10% exosome-depleted FBS (System Biosciences) was used when cells were to be treated with exosomes and particles.

### Human and mouse serum collection

The human study was approved by the Institutional Review Board for the Protection of Human Subjects in Research at the Nagoya City University (approval number: 60-18-0151). Sample collection was carried out in accordance with the approved guidelines. Blood was collected for standard clinical tests at the Nagoya City University Hospital with informed consent and then the mixtures of remaining volumes were kept at 4 °C until use within 2 days. Blood was collected in vessels containing serum-separating agent and processed per the manufacturer’s instructions.

All animal procedures were approved by the National Institute Environmental Studies (NIES) Animal Research Ethics Board following the guidelines of the Japanese Council on Animal Care (approval number: AE-19-34). Whole blood was collected from abdominal vena cava of female C57BL mouse (14 weeks) under deep anesthesia with intraperitoneal administration of pentobarbital sodium. Blood sample without anti-coagulant was centrifuged at 1500*g* for 15 min at 4 °C, and allowed to clot at room temperature for 60 min. After centrifugation at 1500*g* for 15 min at 4 °C, the resulting serum supernatant was stored at – 80  °C until use.

### Exosome isolation and characterization

Human or mouse serum was used for confirmation of the exosome isolation method or cell exposure experiments, respectively. The serum was centrifuged at 2000*g* for 10 min at 4 °C, followed by centrifugation at 10,000*g* for 20 min at 4 °C. An aliquot (0.5 mL) of the resulting supernatant was used for exosome extraction using the ExoQuick exosome extraction kit according to the manufacturer’s instructions. Briefly, ExoQuick was added to the 0.5-mL aliquot of supernatant and the mixture was incubated at 4 °C for 1 h, followed by centrifugation at 1500*g* for 30 min at 4 °C . To avoid contamination by serum proteins, the resulting exosome pellet was washed by resuspension in phosphate buffered saline (PBS) followed by ultracentrifugation at 100,000*g* for 70 min at 4 °C. Portions of the extracted exosome fraction were resuspended in PBS or other appropriate solutions. The existence of exosomes was confirmed by morphological observation using TEM and by assessment of the levels of CD9 and CD63 proteins using western blotting. Size distribution was analyzed by a dynamic light scattering method using a Zeta-potential & Particle Size Analyzer (ELS-Z2, Photo OTSUKA Electronics, Osaka, Japan).

### Western blot analysis

The isolated exosomes were lysed with RIPA buffer containing protease inhibitor, PMSF, sodium orthovanadate and phosphatase inhibitor. We measured protein concentrations using a BCA protein assay kit. Proteins in the lysate were resolved on sodium dodecyl sulfate–polyacrylamide gel electrophoresis (SDS-PAGE) under non-reducing conditions, and electroblotted onto a PVDF membrane. The membrane was blocked with PVDF Blocking Reagent and probed with anti-CD9 or -CD63 antibody, followed by HRP-tagged anti-mouse IgG antibody. The immunoreactions on the membrane were visualized using enhanced chemiluminescence (ECL).

### Transmission electron microscopy

Exosome morphology was investigated using TEM. Approximately 25 µL of the suspension of exosomes in PBS was adsorbed onto a carbon supporting film on a 300-mesh copper grid (Nisshin EM, Tokyo, Japan). Excess solution was removed and the grid was negatively stained with 2% uranyl acetate solution. The samples were observed under TEM (JEM1400plus, JEOL, Tokyo, Japan).

### Fluorescent labelling of exosomes

Exosomes were isolated from mouse serum using the isolation method noted above. The resulting exosome fraction then was washed (to eliminate contamination by serum proteins) by resuspension in PBS followed by a second round of ultracentrifugation. To prepare fluorescent exosomes, the purified exosomes were labeled with PKH26 red- or PKH67 green-fluorescent dye according to the manufacturer’s instructions. Unincorporated dye was removed by passage over an Exosome Spin Column; the resulting eluate then was used for the experiment.

### Cellular association of exosomes and 20-nm nanoparticles

T-REx-CHO-MARCO cells were cultured in a multi-well glass-bottom dish (Matsunami, Osaka, Japan) in the presence or absence of 1 µg/mL tetracycline. After 24 h, CHO-MARCO and CHO-CT cells were exposed to green fluorescent exosomes or 20-nm red fluorescent nanoparticles (positive control). After 6 h of culturing, the cell monolayer was washed twice with pre-warmed HBSS to remove non-adherent nanoparticles and exosomes, and the wash buffer then was replaced with fresh HBSS. Cellular association of the exosomes and nanoparticles was examined by confocal super-resolution microscopy (SpinSR10, Olympus, Tokyo, Japan). The images were acquired and analyzed using the imaging software cellSens (version 1.18, Olympus).

To analyze the fluorescent intensity, T-REx-CHO-MARCO cells were cultured in a 96-well black plate with clear bottom (Corning, NY) in the presence or absence of 1 µg/mL tetracycline. CHO-MARCO and CHO-CT cells were exposed to the red fluorescent exosomes or 20-nm red fluorescent nanoparticles. After 6 h, the cell monolayer was washed 3 times with HBSS and fixed in 4% paraformaldehyde for 10 min. After further washes, the fixed cells were incubated with phalloidin-iFluor 488 Reagent and DAPI for 30 min. The cell monolayer was washed twice with PBS, and the supernatant then was replaced with PBS. The images of cell-associated exosomes and nanoparticles were acquired using an IN Cell Analyzer 6000 (Cytiva). The cell images were obtained with excitation/emission filter combinations of 561/605 nm for red fluorescent dye, 405/455 nm for DAPI, or 488/525 nm for phalloidin. Images were automatically collected from fields positioned at 16 randomly selected locations per individual well. Each cell image was analyzed using image analysis software (In Cell Developer Tool Box 1.7; Cytiva). The following 3 parameters were measured: number of cells, fluorescent area, and fluorescent intensity per individual cell. The total fluorescent intensities per one cell were calculated.

### Localization of exosomes or 20-nm nanoparticles with GFP-MARCO

To study the localization of MARCO alone and in combination with exosomes, GFP-MARCO cells were treated with red fluorescent exosomes or 20-nm red fluorescent nanoparticles for 6 h. The localization was examined by fluorescence microscopy (Axio Obserber.Z1, Carl Zeiss, GmbH, Jena, Germany).

### Localization of fluorescent exosomes and 20-nm red fluorescent nanoparticles

T-REx-CHO-MARCO cells were cultured in a multi-well glass-bottom dish in the presence of 1 µg/mL tetracycline. After 24 h, CHO-MARCO cells were treated with both fluorescent exosomes and 20-nm red fluorescent nanoparticles. After 6 h, the localization was examined by confocal super-resolution microscopy (SpinSR10). The images were acquired and analyzed using the cellSens imaging software. Confocal stacks were imported into the Imaris platform (version 9.2.1, 64-bit version; Oxford Instruments, Abington, UK) for post-acquisition visualization and analysis. The Imaris platform was used to analyze the degree of overlap of two channels, red and green, in all cells of the images. Co-localization was analyzed across the entire confocal stack by measuring the intensity of each label in each voxel. The amount of co-localization was measured by voxels with co-localization and converted to the content.

In other experiments, cells were treated separately with either green fluorescent exosomes or 20-nm red fluorescent nanoparticles (one at a time). Specifically, CHO-MARCO cells were exposed to green fluorescent exosomes or 20-nm red fluorescent nanoparticles for 4 h. After removal of non-adherent exosomes or nanoparticles by washing twice with fresh medium, the cells were further treated with the opposing reagent (20-nm red fluorescent nanoparticles or green fluorescent exosomes, respectively) for 4 h. The cells then were washed twice with HBSS and observed using confocal super-resolution microscopy (SpinSR10).

### Uptake inhibition study

T-REx-CHO-MARCO cells were cultured with 1 µg/mL tetracycline in a 96-well black plate with clear bottom. CHO-MARCO cells were precultured with or without 10 µM cytochalasin D, 50 µM dynasore, 5 µg/mL MβCD, 20 µM LY294002, 10 µM nocodazole, or 0.1% DMSO (as a control) in fresh medium containing 10% exosome-depleted FBS. After 1 h, the cells were exposed for 6 h to red fluorescent exosomes, or to 20- or 200-nm red fluorescent particles, in the presence of the same respective inhibitor. The cell monolayer was washed 3 times and fixed with 4% paraformaldehyde for 10 min. After the washing, the fixed cells were stained with DAPI. The cell monolayer was washed twice with PBS and the rinse buffer was replaced with PBS. The cellular association with exosomes or 20- or 200-nm fluorescent particles was observed by fluorescence microscopy (Axio Obserber.Z1).

For inhibitory experiment by MARCO ligand, CHO-MARCO cells were precultured with 200 µg/mL polyG, polyC, or 1% endotoxin-free sterile water (solvent for polyG and polyC, as a control) for 30 min. The cells were exposed to red fluorescent exosomes for 4 h in the presence of the same respective ligand. After incubation, the method was the same as described above.

### Statistical analysis

Data are presented as mean ± SEM. Statistical analyses were performed by ANOVA followed by Bonferroni’s post hoc analysis. The statistical significance level was set at *p* ≤ 0.05.

## Supplementary Information

Supplementary Legends.

Supplementary Figures.
