# Supplementary material for: Scavenger receptor MARCO contributes to cellular internalization of exosomes by dynamin-dependent endocytosis and macropinocytosis
Source: Sci Rep. 2020 Dec 11;10:21795. doi: 10.1038/s41598-020-78464-2 (PMC7733512; doi:10.1038/s41598-020-78464-2)
Supplement: Supplementary file 1 — Supplementary Legends. [file 41598_2020_78464_MOESM1_ESM.docx]

**Supplemental Fig. 1. The full-length blots of Fig. 1A.**

The lysate of the resulting exosome fraction was resolved by SDS-PAGE and electroblotted onto a PVDF membrane. The blot was probed with anti CD9- and CD63-antibodies, followed by a corresponding HRP-tagged secondary antibody.

**Supplemental Fig. 2. Fluorescence microscopic analyses of the exosomes (A) or 20-nm particles (B) associated with CHO-CT or CHO-MARCO cells.**

T-REx-CHO-MARCO cells were cultured in a 96-well black plate with clear bottom with (CHO-MARCO) or without (CHO-CT) tetracycline. After 24 hours of culture, the cells were incubated to red fluorescent exosomes (A) or 20-nm fluorescent nanoparticles (B) for 6 hours. After incubation, the cells were washed twice with HBSS and fixed with formaldehyde, followed by staining with DAPI and phalloidin. The cellular association was observed by fluorescence microscopy. These cell monolayers were used for quantification analyses.

**Supplemental Fig. 3. Localization of MARCO and exosomes (a) or 20-nm nanoparticles (b) in MARCO-GFP cells.**

MARCO-GFP cells were incubated with red fluorescent exosomes (a) or 20-nm red fluorescent nanoparticles (b) for 6 hours. The localization of MARCO and exosomes or 20-nm nanoparticles was examined by fluorescence microscopy. The exosomes and nanoparticles also were observed on the ruffling membrane, as indicated here by arrows.

**Supplemental Fig. 4. Localization of exosomes or 20-nm nanoparticles in CHO-MARCO cells.**

T-REx-CHO-MARCO cells were cultured in a multi-well glass-bottom dish in the presence of tetracycline. After 24 hours of culture, the cells were incubated with 20-nm red fluorescent nanoparticles (a) or green fluorescent exosomes (b) for 4 hours. After washing of cell monolayers twice with fresh medium, the respective cells were further treated with fluorescent exosomes (a) or 20-nm fluorescent particles (b) for 4 hours. (c): Thus, the cells were treated (separately) with fluorescent exosomes and 20-nm fluorescent nanoparticles over a total of 8 hours. The cells were washed twice and observed using confocal super-resolution microscopy.
