# Supplementary material for: Scavenger receptor MARCO contributes to cellular internalization of exosomes by dynamin-dependent endocytosis and macropinocytosis
Source: Sci Rep. 2020 Dec 11;10:21795. doi: 10.1038/s41598-020-78464-2 (PMC7733512; doi:10.1038/s41598-020-78464-2)
Supplement: Supplementary file 2 — Supplementary Figures. [file 41598_2020_78464_MOESM2_ESM.pdf]

Scavenger receptor MARCO contributes to cellular internalization of exosomes by dynamin-dependent endocytosis and macropinocytosis

Sanae Kanno[a]*, Seishiro Hirano[b], Tsubasa Sakamoto[a], Akiko Furuyama[b], Hiroshi Takase[c], Hideaki Kato[a], Mamiko Fukuta[a], Yasuhiro Aoki[a]

[a] Department of Forensic Medicine, Nagoya City University Graduate School of Medical Sciences, 1 Kawasumi, Mizuho-cho, Mizuho-ku, Nagoya 467-8601, Japan

[b] Center for Health and Environmental Risk Research, National Institute for Environmental Studies, 16-2 Onogawa, Tsukuba, Ibaraki 305-8506, Japan

[c] Core Laboratory, Nagoya City University Graduate School of Medical Sciences, 1 Kawasumi, Mizuho-cho, Mizuho-ku, Nagoya 467-8601, Japan

*Correspondence should be addressed to: Sanae Kanno, Ph.D.

Department of Forensic Medicine, Graduate School of Medical Sciences, Nagoya City University, 1 Kawasumi, Mizuho-cho, Mizuho-ku, Nagoya 467-8601, Japan

Tel: +81-52-853-8180

E-mail: sanae@med.nagoya-cu.ac.jp

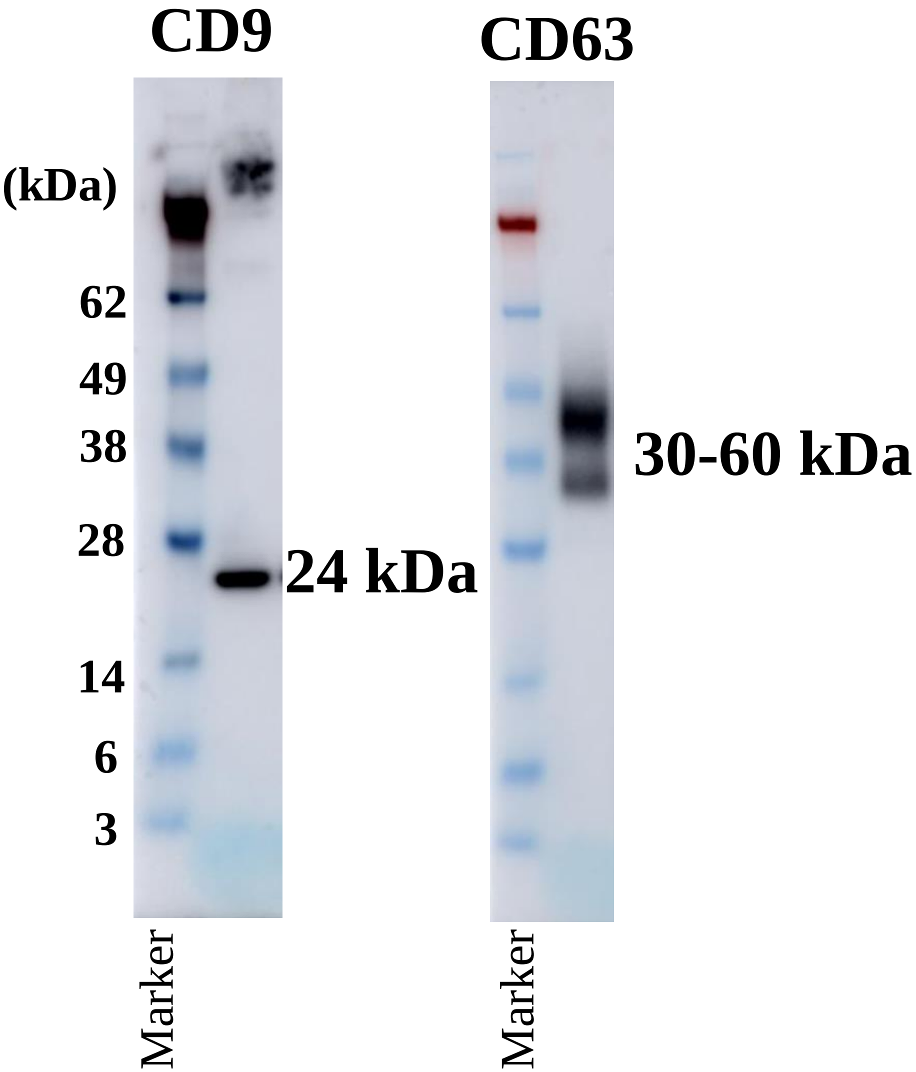

Supplemental Fig. 1

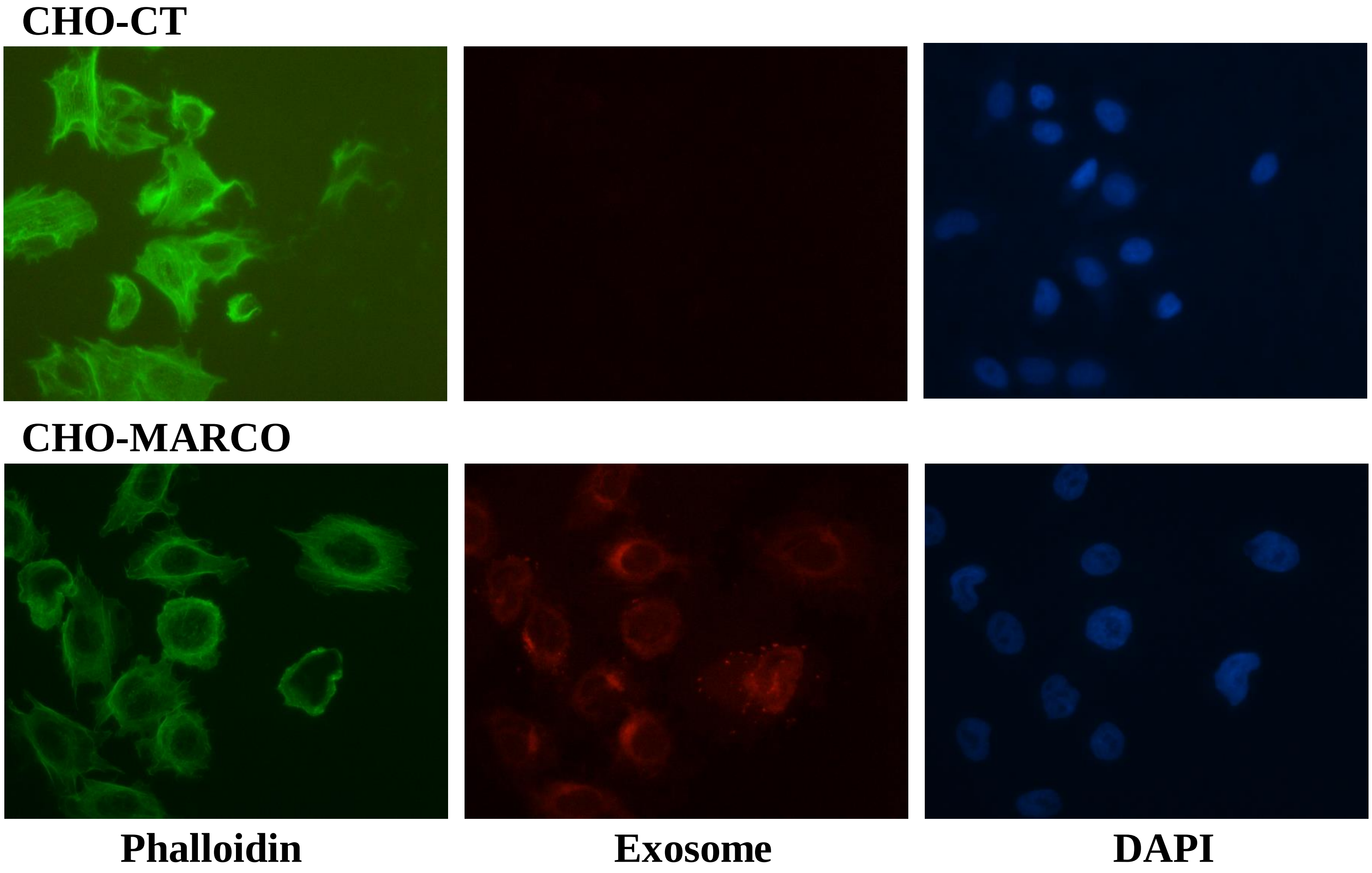

Supplemental Fig. 2A

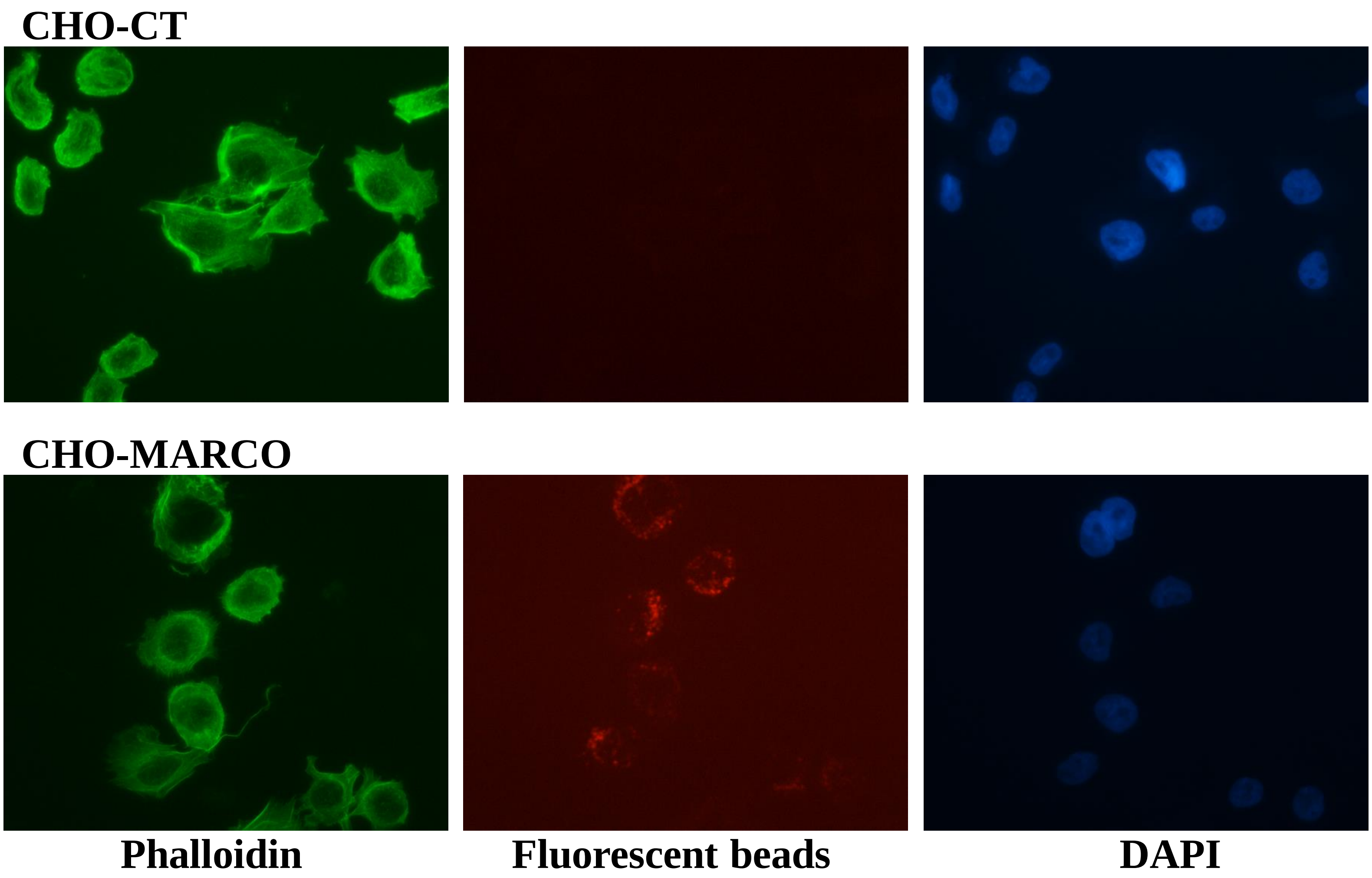

Supplemental Fig. 2B

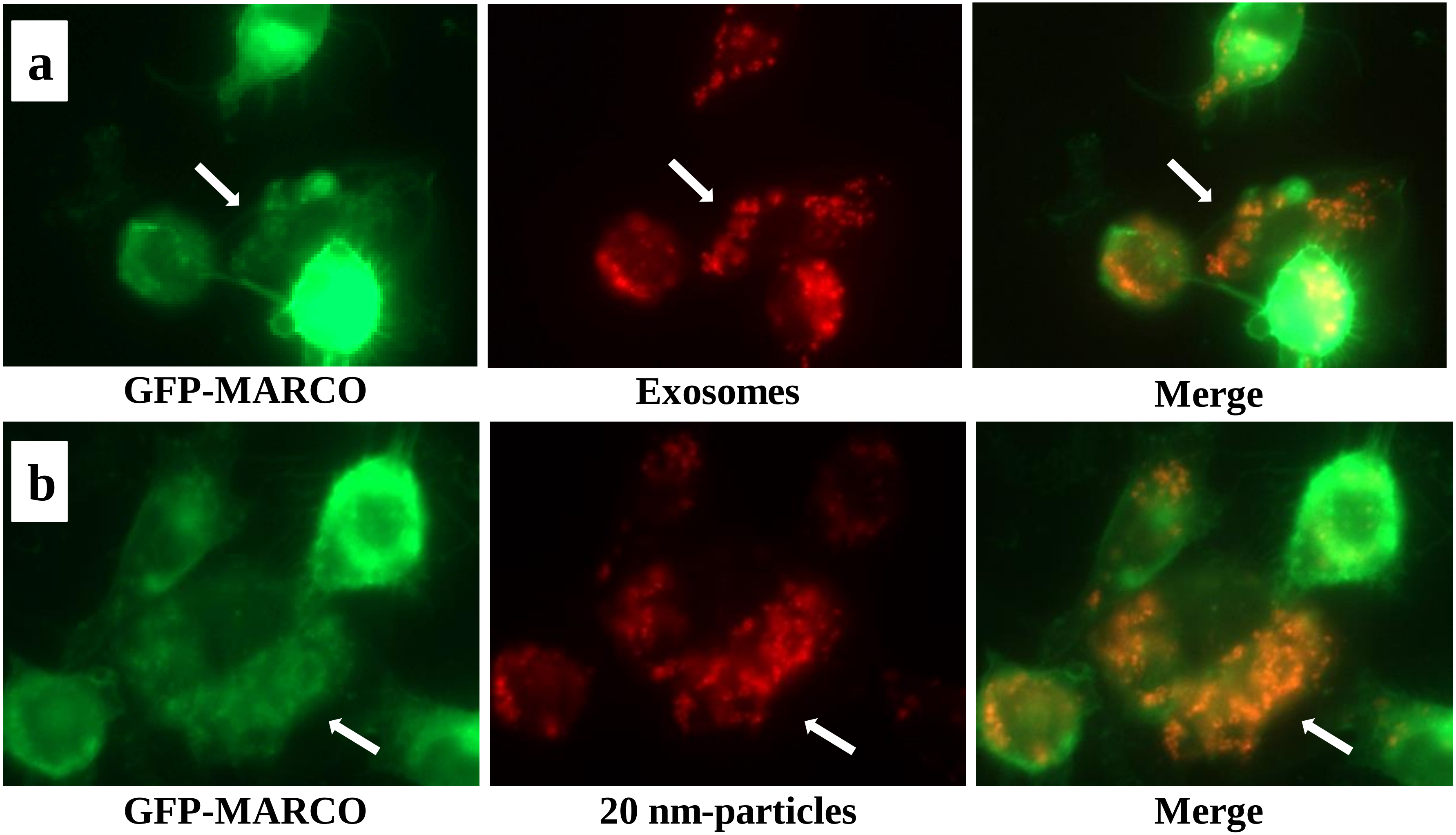

Supplemental Fig. 3

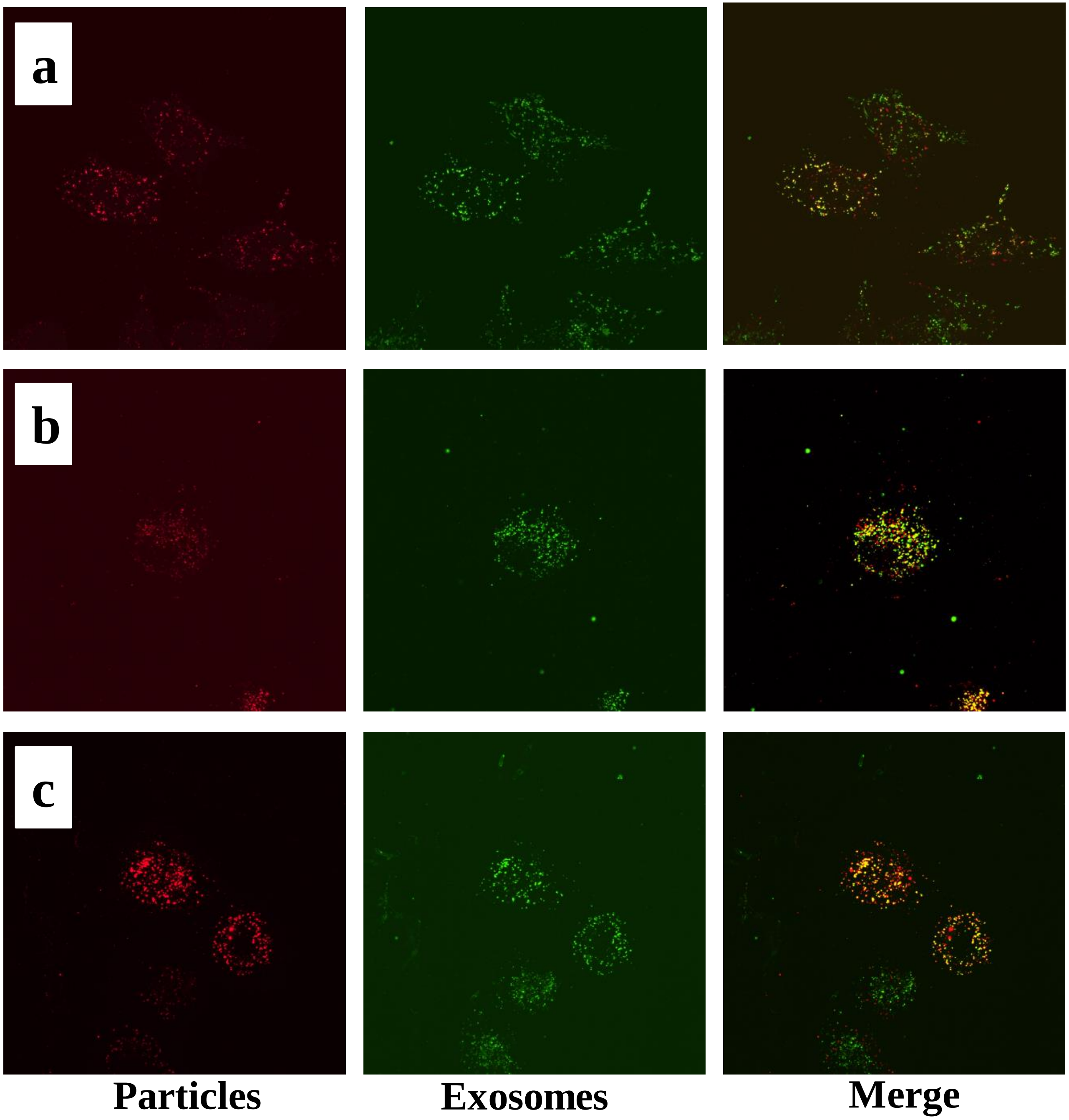

Supplemental Fig. 4
